# Singapore's COVID‐19 Genomic Surveillance Programme: Strategies and Insights From a Pandemic Year

**DOI:** 10.1111/irv.70060

**Published:** 2024-12-19

**Authors:** Hao Yi Tan, Nur Huda Khamis, Alvin Goh, Tania K. L. Mah, Benny Yeo, Jie Yin Ngan, Yichen Ding, Cui Lin, Sae‐Rom Chae, Phoebe Lee, Zheng Jie Marc Ho

**Affiliations:** ^1^ Communicable Diseases Group Ministry of Health Singapore; ^2^ Health Systems Group Harvard T.H. Chan School of Public Health Boston Massachusetts USA; ^3^ National Public Health Laboratory Ministry of Health Singapore; ^4^ Asia Centre for Health Security, Saw Swee Hock School of Public Health National University of Singapore Singapore

**Keywords:** Acute Respiratory Infections (ARI), COVID‐19, Emerging infectious diseases, Genomic Surveillance, Health Strategy, Pandemic preparedness, Singapore, Whole genome sequencing (WGS)

## Abstract

**Background:**

During the COVID‐19 pandemic, genomic surveillance was crucial for monitoring virus spread and identifying variants. Effective surveillance helped understand transmission dynamics. Singapore had success in combating COVID‐19 through its surveillance programmes. This paper outlines Singapore's strategy and its impact on public health during the transition to endemicity over 54 weeks from February 2022 to February 2023.

**Methods:**

In May 2022, Singapore expanded its acute respiratory infections (ARI) surveillance to enhance COVID‐19 detection. COVID‐19–positive samples from ARI cases were sent to the National Public Health Laboratory for whole genome sequencing (WGS). WGS data informed public health actions based on transmission origins and case severity.

**Results:**

Over 54 weeks, NPHL sequenced 18,918 (73%) samples. Analysis showed 29% imported and 71% local cases. Severe cases accounted for 12% and were mostly elderly, specifically those aged 80 years old and above. Variant analysis identified 11 predominant variants and 288 subvariants. Omicron BA.2, BA.5 and XBB were initially dominant, followed by increased variant heterogeneity. Severe cases mirrored these trends.

**Conclusion:**

Genomic surveillance was integral in Singapore's COVID‐19 response, guiding timely public health decisions. Effective variant tracking supported proactive measures. The experience underscores the importance of genomic surveillance for future pandemic preparedness and emerging disease detection, emphasising its role in shaping pandemic responses and global health.

## 1 Introduction

Throughout the COVID‐19 pandemic, pathogen genomics proved to be an effective tool to monitor its spread locally and globally, providing guidance for public health interventions [1]. As the virus mutated, it gave rise to variants with different characteristics in immune escape, infectivity and virulence [2]. These led to multiple waves of COVID‐19 infections [3]. Genomic surveillance played a key role in the detection, characterisation and tracking of such emerging variants [4].

Globally, genomic surveillance ranges from global and regional efforts to national initiatives, smaller subnational areas to none at all. Genomic surveillance was more effective when countries shared information as part of larger networks and accumulating sufficient data to accurately observe and even predict trends [5]. Furthermore, effective methods typically involve a comprehensive process beyond just sample collection and genotyping, but also linking it with metadata and sharing information with relevant authorities for the implementation of appropriate public health measures [6]. Thus far, only a handful of countries have published information on their genomic surveillance approaches [7, 8, 9, 10, 11].

Singapore is a city‐state with a high population density and a hub for international trade and travel. It has also had one of the lowest COVID‐19 mortality rates worldwide [12]. In this article, we outline Singapore's country‐wide COVID‐19 genomic surveillance programme and the role it played in informing public health policies and actions during the pandemic. We also describe outcomes across 54 weeks (from 1 February 2022 to 11 February 2023, the latter of which was the last week before Singapore moved from a pandemic state to endemicity).

## 2 Methods

### 2.1 Overview of Surveillance of Respiratory Infections

Surveillance of respiratory infections in Singapore is overseen by Singapore's Ministry of Health (MOH) and supported by the country's regional health systems, private clinics and the National Public Health Laboratory (NPHL). Surveillance for influenza‐like illnesses (ILI)—defined as fever ≥ 38.0°C accompanied by cough, regardless of duration—was established long before the COVID‐19 pandemic. Samples were collected from selected government primary care clinics and private general practitioner (GP) clinics.

In May 2022, the ILI programme underwent significant expansion in an attempt to improve surveillance of COVID‐19 and other respiratory illnesses. This involved the sampling of Acute Respiratory Infection (ARI) cases—defined as cough, sore throat, runny nose and/or fever. The impetus was the need to monitor circulating respiratory viruses in the community and the periodic waves they caused. The more stringent ILI criteria would not have captured many viruses of importance, including COVID‐19. Since then, the MOH has been able to monitor the trends of COVID‐19, influenza, respiratory syncytial virus and other respiratory pathogens within an increased number of community samples.

The strategic deployment of the surveillance programme across both private and government primary care clinics across the island played a pivotal role in ensuring a robust and representative sample of the population seeking medical care for respiratory symptoms. This enabled the MOH to gain a comprehensive overview of the prevalence and trends of respiratory pathogens within the community.

Specific to COVID‐19, positive samples collected underwent whole genome sequencing (WGS). The information gleaned was then used for sense‐making, including anticipating oncoming waves and facilitating changes to public health measures and preparing healthcare treatment capacities as necessary.

From the first imported COVID‐19 case in Singapore on 23 January 2020 to February 2023, all medical practitioners and diagnostic laboratories were legally required, under the Infectious Diseases Act, to notify the MOH of individuals who tested positive for COVID‐19, including by polymerase chain reaction (PCR) or antigen rapid tests (ART). However, after large COVID‐19 Delta and Omicron waves from September to November 2021 and January to March 2022, respectively, it was observed that case ascertainment had likely fallen significantly, including from self‐testing using readily available commercial ART kits and reduced testing by doctors.

### 2.2 Sampling for COVID‐19 Surveillance

**FIGURE 1:**
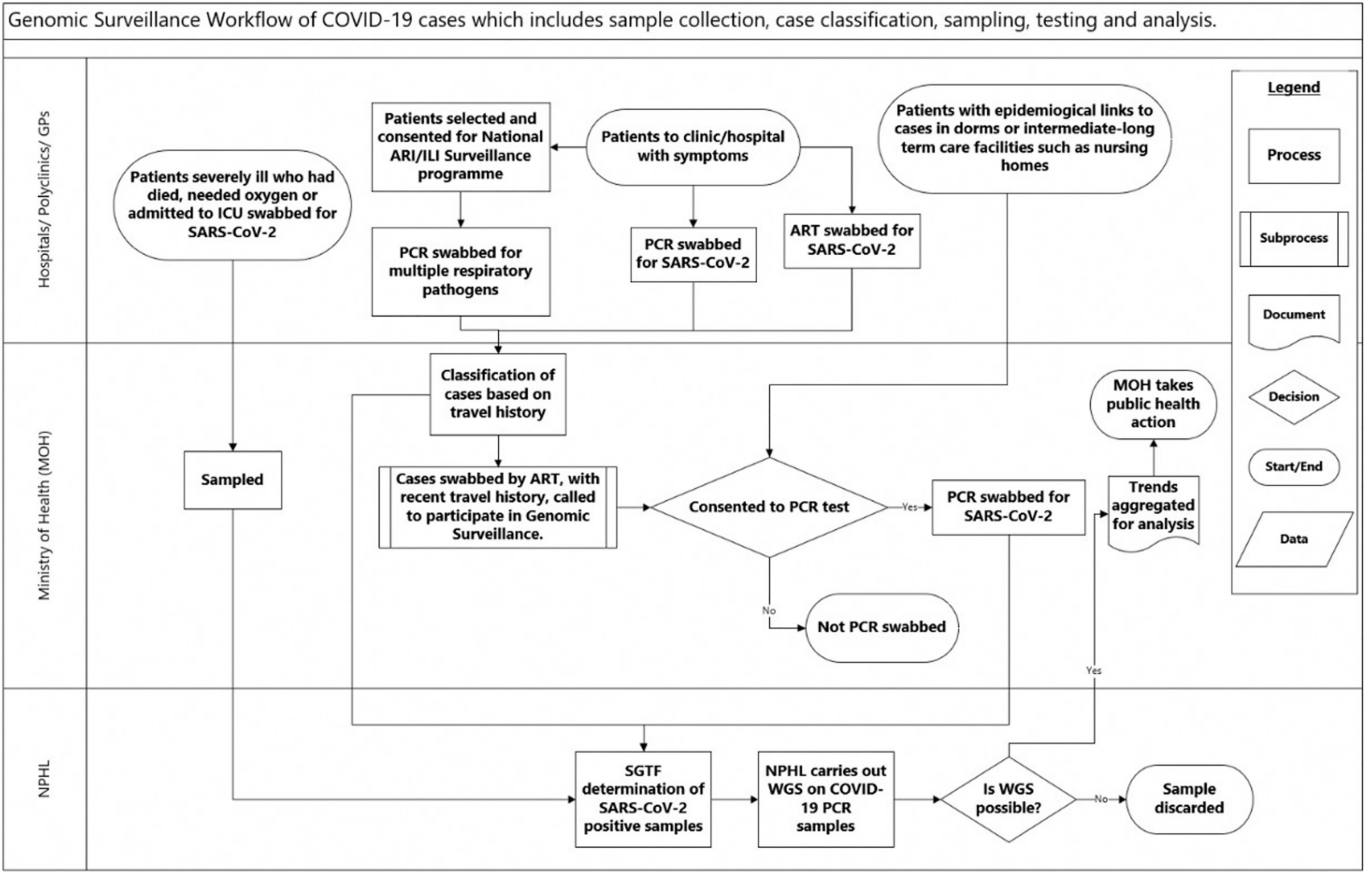
Workflow of sampling imported, community and severe COVID‐19 cases for genomic surveillance.

With surveillance systems' enhancements made in May 2022, samples were collected from cases presenting with ARI symptoms through an established workflow (see Figure 1). They fell into the following groups:

#### 2.2.1 Recent Travelers

Individuals with overseas travel in the past 5 days who tested positive on PCR or ART were notified to the MOH. The latter group was sent for a follow‐up nasopharyngeal PCR swab, which was sent to NPHL for COVID‐19 testing and subsequent WGS. Samples were taken out of the total pool of travellers and took into consideration source countries. The sampling strategy prioritised non‐Asian countries, as cases from these regions were relatively rare. There was at least 10% of cases from Asia in the sample, prioritised countries with limited sequencing capabilities and of those reporting emerging or ongoing outbreaks.

#### 2.2.2 Community

Patients presenting with ARI symptoms at eligible clinics were randomly selected by primary care doctors and offered free PCR testing. Samples collected were dispatched to NPHL for testing across a range of respiratory pathogens, including for SARS‐CoV‐2, the pathogen causing COVID‐19.

#### 2.2.3 Targeted Surveillance

In addition to surveillance among the general population, targeted surveillance was conducted for two groups of individuals. One was among migrant workers living in dormitories who had an increased risk of outbreaks due to congregate living environments, and the other was among nursing home residents, who were typically elderly (mostly more than 80 years old) Singapore residents at higher risk of severe infection due to advanced age and the presence of medical comorbidities. Outbreaks within these higher risk populations serve as valuable surveillance indicators. These settings present a higher risk of infection compared to the general community and thereby serve as possible early indicators of potential community transmission.

#### 2.2.4 Selection for WGS

Of these COVID‐19 positive cases, a subset was selected for WGS. The MOH preferentially selected patients with severe disease and imported cases from countries reporting outbreaks of emerging COVID‐19 variants.

### 2.3 Laboratory Testing and WGS

The NPHL performed sample extraction and RT‐PCR on swab samples, including those detected of SARS‐CoV‐2 antigen via ART. Upon detection of SARS‐CoV‐2 RNA in the samples, Taqpath COVID‐19 RT‐PCR was performed to determine the presence of S‐gene target failure (SGTF). SGTF was used as a surrogate laboratory marker in order to speed up detection of certain variants as the time required for this result to be produced was 2–7 days, less than half the time it took for a WGS result. A subset of the SARS‐CoV‐2–positive samples was selected for COVID‐19 WGS based on the prevailing ratio of SGTF of that particular epidemiology week. For WGS, cDNAs were synthesised, and amplicon‐based amplification was performed with primers tiled across the whole SARS‐CoV‐2 genome (ARTIC V4.1) to form multiple DNA fragments of base pairs. These fragments are further processed by tagmentation and PCR before samples were loaded in the Illumina MiSeq system for WGS. Raw NGS reads were trimmed and mapped to the original Wuhan reference genome (accession number NC_045512) by bwa mem. Variant calling and consensus sequence generation were performed by bcftools. Lineage calling and variants detection were performed by the Pangolin web application (pangolin.org‐uk.io). Variants detected were cross‐checked with Nexclade and CoVsurver database. All lineages identified as recombinant lineages/variants by Pangolin were referred to recombinants in the figure.

Quality control procedures for SARS‐CoV‐2 WGS were applied through the assessment of depth, completeness and mixed sites. When mixed sites were detected, the NPHL did a manual review of the result before generating consensus of the variant type. Data were then shared with open access databases such as Global Initiative on Sharing All Influenza Data (GISAID).

### 2.4 Analysis and Assessment

Upon receipt of WGS results, the Ministry of Health (MOH) analysed the data, correlated the information obtained with other surveillance data such as wastewater test results and decided on follow‐up public health actions as necessary. Due to the large number of subvariants from the various sublineages, variants were grouped based on their parental lineages before being reported in datasets. WGS data were collated and assessed routinely each week by epidemiologists and public health officers for public health actions.

Associated cases were classified primarily based on origins of virus transmission, categorised as imported or local. ‘Imported cases’ comprised of individuals who contracted COVID‐19 overseas and then travelled to Singapore. ‘Local cases’ comprised of individuals likely infected while in Singapore. Subsequently, severity was considered and extracted as a sub‐group known as ‘Severe cases’. These were a subset of local or imported cases who needed oxygen supplementation, admission to the intensive care unit or died.

## 3 Results

From February 2022 to February 2023, a total of 17,496 community ARI and ILI samples were received from the National ARI/ILI programme, out of which 4250 were included in the genomic surveillance. With the inclusion of samples from different sources, a total of 25,770 COVID‐19 positive cases were sampled for the genomic surveillance programme. Out of these, 18,918 (73%) were successfully sequenced from 1 February 2022 to 11 February 2023. Between 400 and 500 SARS‐CoV‐2–positive samples were sequenced by NPHL per week. Following analysis, 411 of which were excluded from further analysis due to incomplete data (e.g., demographic data). The remaining 18,507 (71%) samples were analysed.

### 3.1 Demographics

Of 18,507 samples, 5384 (29%) were from imported cases, and the rest were local cases (refer to Table 1 below). Two thousand two hundred four (12%) of all samples were from severe cases (imported and local). Fifty‐three percent of all samples were male. For ethnicity, 62% of cases were registered as being of ethnic Chinese descent, with individuals of Malay descent constituting 13% of the cases, while those of Indian descent accounting for 15%. The remaining 10% were a mix of other racial groups.

**TABLE 1:** Demographic analysis of all successful WGS results from 1 February 2022 to 11 February 2023.

	Imported	Local	Severe (subset of either imported or community)	Total (imported + community)
Total number	5384	13,123	2204	18,507
Gender				
Male	2964 (55%)	6873 (52%)	1229 (56%)	9837 (53%)
Female	2420 (45%)	6250 (48%)	975 (44%)	8670 (47%)
Age				
< 18	179 (3%)	818 (6%)	23 (1%)	997 (5%)
18–30	1206 (22%)	1702 (13%)	12 (1%)	2908 (16%)
31–40	1509 (28%)	1625 (12%)	20 (1%)	3134 (17%)
41–50	1093 (20%)	1180 (9%)	57 (3%)	2279 (12%)
51–60	747 (14%)	1288 (10%)	151 (6%)	2035 (11%)
61–70	400 (8%)	1653 (13%)	394 (18%)	2053 (11%)
71–80	159 (3%)	2056 (16%)	628 (28%)	2215 (12%)
> 80	91 (2%)	2801 (21%)	919 (42%)	2892 (16%)
Ethnicity				
Chinese	2887 (54%)	8636 (66%)	1636 (74%)	11,523 (62%)
Malay	573 (11%)	1853 (14%)	356 (16%)	2426 (13%)
Indian	916 (17%)	1876 (14%)	184 (9%)	2792 (15%)
Others	1008 (18%)	758 (6%)	28 (1%)	1766 (10%)

Cases were distributed across various age groups, with the most represented ages being 31–40, 18–30 and > 80, constituting 17%, 16% and 16% of the total cases, respectively. Unsurprisingly, the bulk of severe COVID‐19 cases was elderly. Those 60 years old and above made up 88% of all severe cases. This is despite this age group making up only 50% of local cases and 12% of imported cases among the study population.

### 3.2 Variants and Trends Over Time

Over 54 weeks, 11 predominant variants were detected, yielding 288 distinct subvariants.

When comparing the three populations—imported, local and severe cases—it was observed that the emergence of subvariants may occur at similar or different times across these groups. Some subvariants manifested within the same week across all populations, while others initially appeared in either the imported or local populations (or both together) before being identified among severe cases. This pattern was observed for BA.4, BN.1, XBB, BQ.1x and CH.1.1, suggesting a sequential introduction within the population of travellers, followed by emergence into the local population, and eventually affecting the severity of cases.

In terms of predominance, earlier trends demonstrated greater homogeneity, with a single variant swiftly emerging as the predominant circulating strain, a contrast to the heterogeneous blend of subvariants observed later.

Among imported cases, the Omicron BA.2 variant predominated from February to May 2022, succeeded by the dominance of the Omicron BA.5 variant from June to September 2022. From October 2022 to February 2023, the rapid emergence of new variants resulted in a mixture of detected variants, including XBB, BA.5, BQ.1, BN.1 and CH.1.1.

**FIGURE 2:**
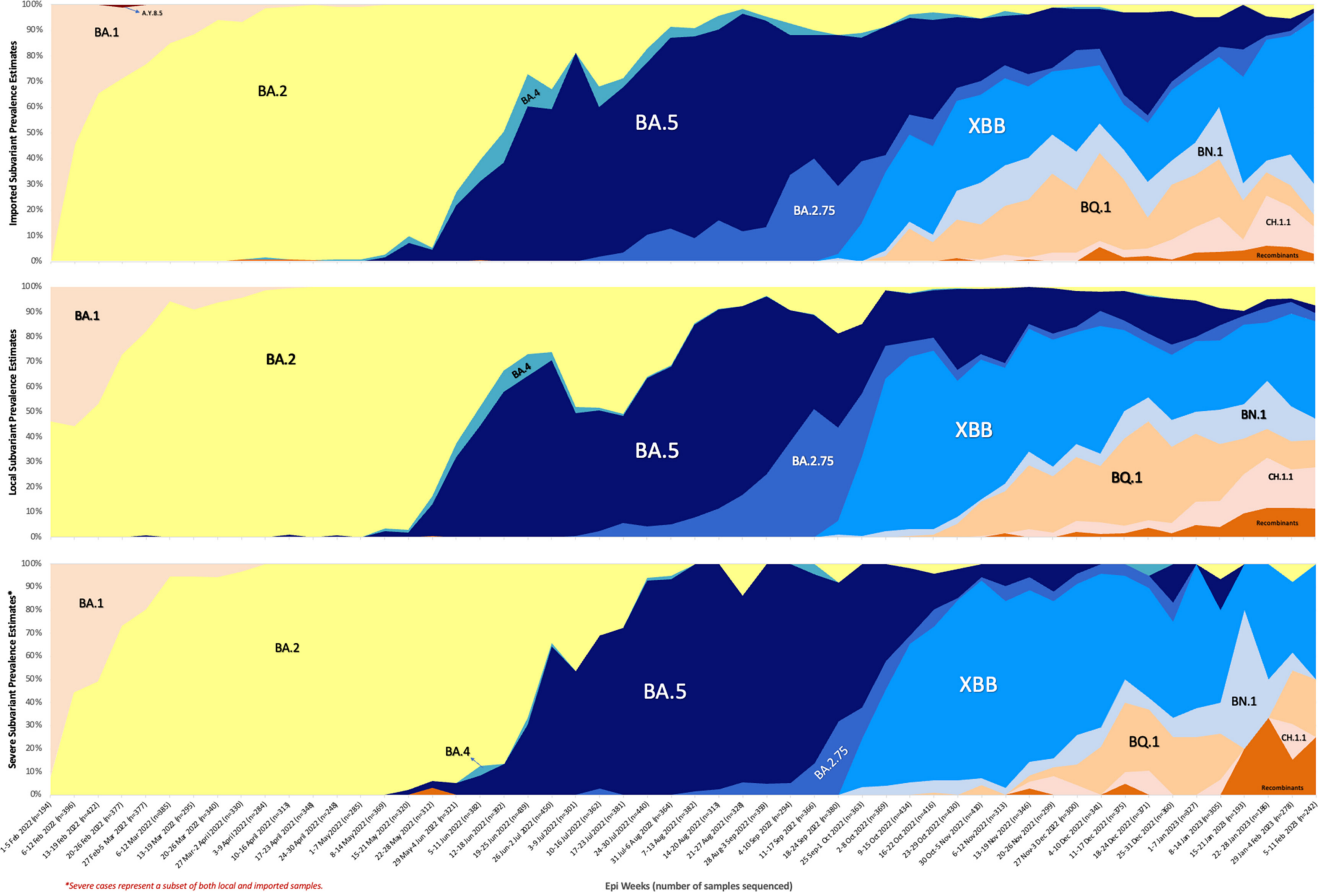
Area chart plotting the variant distribution of imported, local and severe COVID‐19 cases from 1 February 2022 to 11 February 2023.

**FIGURE 3:**
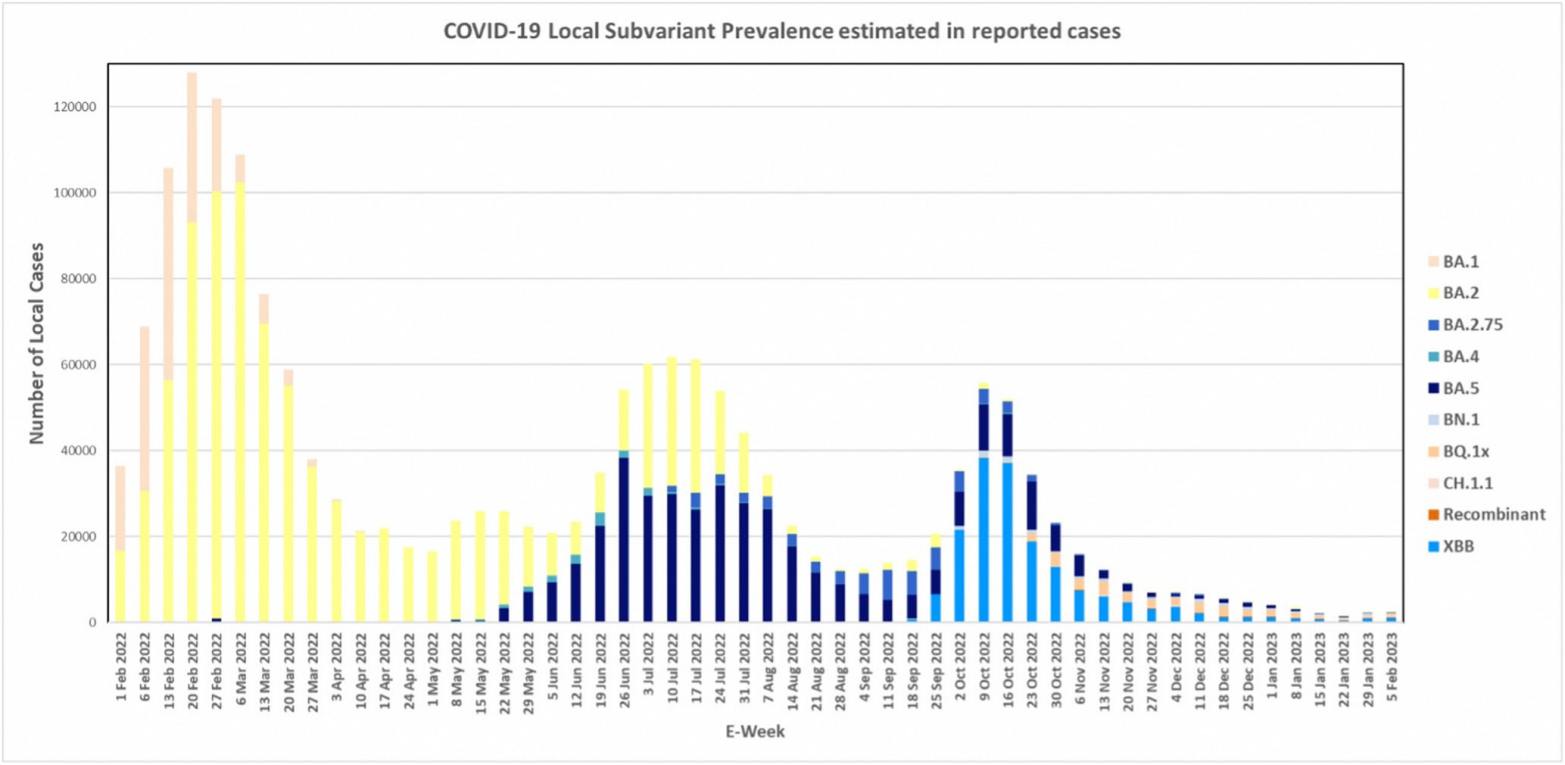
Bar chart plotting the number of reported COVID‐19 cases in Singapore from 1 February 2022 to 11 February 2023, subdivided into variant proportions by colour.

Following a similar trajectory and close in timing as imported cases, the Omicron BA.2 variant emerged as the predominant variant circulating among local community cases in Singapore from February to June 2022. Subsequently, it was replaced by Omicron BA.5 from July until September 2022, as depicted in Figure 2 (area chart). Of note, the ascendancy of a single variant often coincided with a surge in COVID‐19 cases. This can be seen clearly in Figure 3, where the uptick in cases accompanies the increasing dominance of a single variant. Following the XBB wave, a marked increase in heterogeneity was observed in the variant pool, with multiple different variants detected in samples collected from COVID‐19 cases in Singapore from November 2022 onwards.

A comparable trend was observed in severe cases of COVID‐19, with the dominance of single variants early in the pandemic giving way to significantly more heterogeneity later on.

Notably, the trends of variant emergence in Singapore largely mirrored global patterns [13], with sequential waves of predominant variants such as Delta, Omicron BA.2 and BA.5. As a major travel hub, Singapore detected introductions of new variants soon after they appeared globally, similar to other highly connected countries.

### 3.3 Targeted Surveillance

The primary objective of targeted surveillance was to monitor outbreaks within higher risk populations, with the working hypothesis that these could act as early indicators of broader community transmission. However, our findings showed that outbreak patterns within these groups typically aligned with, rather than preceded, trends observed in the general community. Despite this, targeted surveillance has proven valuable for investigating specific outbreaks, particularly by providing insights into transmission dynamics within clusters, such as identifying secondary variant introductions and situations involving multivariant transmission. In our analysis, cases from targeted surveillance were generally included in the overall community dataset; however, cases with a recent travel history were classified as imported.

## 4 Discussion

### 4.1 Usefulness of Genomic Surveillance in Tracking Emergence and Spread of COVID‐19 Variants

Globally, genomic surveillance of COVID‐19 has been instrumental in navigating the challenges posed by the unprecedented scale and dynamics of the pandemic [6]. Like many major cities in the world, Singapore is a densely populated and globally connected travel hub with a high risk of communicable disease outbreaks [14]. The acquiring of COVID‐19 genomic data, as well as the subsequent processing and sharing of such data, has proved useful in guiding the nation through the pandemic. Besides guiding national policy, sharing of genomic information with the global community through organisations such as GISAID helped to track the emergence and spread of variants internationally, enabling a coordinated global response [15].

In Singapore, we observed through our genomic data that earlier in the pandemic that each wave was primarily driven by a single COVID‐19 variant that exceeded its counterparts in terms of its transmissibility. This was seen by directly correlating changes in variants over time with the total COVID‐19 cases in the community as well as those who were hospitalised. This gave Singapore's MOH greater visibility into when waves were occurring or likely to occur, and if one or more variants were driving the wave. Since this pattern was repeated, continuous genomic surveillance gave the MOH the opportunity to be better prepared when a single variant started to become more predominant in community cases, severe cases or both. Similar to many other countries, the early indication allowed the government to consider taking public health actions in advance [6, 16]. For example, at the beginning of the BA.2, BA.5 and XBB waves, the MOH was able to adjust public health and social measures and issuing public advisories to slow spread, or activating hospital surge capacity to meet anticipated caseloads.

Genomic surveillance for travellers into Singapore was also useful in monitoring the influx of variants into Singapore. The programme allowed for visualisations of up‐to‐date information regarding variants being imported from any given country. Although not implemented, this would have allowed the government to take specific border control measures for travellers from areas where a new variant of concern was emerging.

### 4.2 Assessing Impact of Variants

Genomic surveillance was especially useful in detecting variants with significantly different transmissibility and severity than expected. Since the start of the pandemic, Singapore used its genomic data in tandem with other surveillance data for modelling purposes to determine how large each wave could end up being and how long the government would have to maintain infection control and hospital surge capacity measures [17].

For instance, the XBB wave hit Singapore in October 2022. The variant was highly infectious [18], causing a severe spike in numbers of people who contracted the virus. However, surveillance was able to discern that though XBB was highly infectious, cases with XBB had less severe disease compared to cases with the Delta variant [19, 20]. Furthermore, it was observed that after a rapid rise as the dominant circulating strain in Singapore, its prevalence rapidly declined, and the wave subsided. Just before this wave, the Singapore government had relaxed its mask‐wearing rules, requiring them only in indoor spaces [21]. The fear that the XBB wave could cause a deadly wave placed pressure on the MOH to reinstate compulsory masking in all settings. However, they were able to maintain the gradual relaxing of rules due to surveillance information provided by both genomic surveillance and other complementary epidemiological data.

Similarly, when the People's Republic of China ended its strict zero‐COVID policy in December 2022 [22], many countries imposed border measures on Chinese travellers for fear of importation of new or deadly SARS‐CoV‐2 variants into the country [23]. Singapore chose not to do so [24], as close monitoring of genomic surveillance and public health intelligence found an absence of any extraordinarily infectious or deadly variants. Furthermore, the strains detected from imported cases from China were found to be already in local circulation and commonplace in Singapore. As such, the detailed surveillance allowed MOH to decide that there was little risk of allowing the Chinese travellers into the country since they were not introducing any new variants. This meant that Singapore could avoid potentially costly and unpleasant border measures on travellers while remaining moderately confident of the decision's impact on the country's healthcare system. Ultimately, the influx of these travellers entering Singapore did not cause any COVID‐19 waves nor an overwhelming of the healthcare system.

Although vaccination strategy is not the primary focus of this paper, it is worth noting that Singapore maintained high COVID‐19 vaccination coverage throughout the pandemic [25]. This strategy of widespread and early vaccination focused on high‐risk populations likely played a critical role in mitigating the severity of variant‐driven outbreaks, as seen in the relatively low rates of severe cases and hospitalisations despite the emergence of transmissible variants. Furthermore, other publications have highlighted the effectiveness of vaccinations and boosters in the prevention of severe COVID‐19 cases in Singapore [26, 27]. High vaccination coverage, including with boosters, may have contributed to cushioning the impact of new variants, helping to keep the healthcare system from becoming overwhelmed.

### 4.3 Other Uses of Genomic Surveillance

Exploration of additional applications of genomic surveillance, such as wastewater testing in the community and in migrant worker dormitories, as well as sewage water from commercial planes at airports, was started in the pandemic [28] and continues to be developed. For example, Singapore was able to use wastewater testing effectively in certain situations throughout the pandemic, such as in migrant worker dormitories, student accommodations or even entire geographical localities [29, 30, 31]. Going forward, such applications are well placed to detect future spikes especially if community testing rates are low. This allows for detection of variants of concern before healthcare services are overwhelmed.

Genomic surveillance is already used in many diseases worldwide, such as for influenza and tuberculosis. With newer genomic technologies on the rise, there is potential to increase the scope and scale of genomic surveillance especially in areas such as antimicrobial resistance [32]. A comprehensive genomic surveillance programme at the national or regional level would enhance prevention and preparedness for future pandemics, and implementing metagenomics as a surveillance tool enables health authorities to monitor emerging infectious diseases, including Disease X [33, 34]. Therefore, establishing robust infrastructure and processes for genomic surveillance ensures rapid detection and response, thereby mitigating the impact of future pandemics.

### 4.4 Unique Elements of Singapore's Genomic Surveillance Approach

Singapore's genomic surveillance for COVID‐19 embodies several unique elements that distinguish it from efforts in other countries, such as the United Kingdom and the United States. While many nations implemented genomic surveillance, Singapore's approach demonstrated innovations that were suited to its high‐density, high‐travel environment, as well as its emphasis on comprehensive public health coordination of different surveillance modalities.

First, Singapore's programme was designed to integrate COVID‐19 surveillance with pre‐existing respiratory infection monitoring systems and expand them from within. This strategy differs from countries such as the United Kingdom and the United States, which successfully developed separate, large‐scale COVID‐19 genomic surveillance programmes in response to the pandemic [35, 36]. Singapore leveraged its existing ILI and ARI surveillance infrastructure, allowing the MOH to pivot quickly and expand surveillance to include COVID‐19 and other respiratory pathogens. By maintaining this integrated framework, Singapore has been able to adapt its surveillance systems beyond the COVID‐19 pandemic, into an approach that is also suitable for long‐term pandemic preparedness.

Another distinctive aspect of Singapore's strategy was its targeted sampling approach, which was centrally coordinated by the MOH. Singapore purposefully biased sampling towards high‐risk groups, including recent travellers, dormitory residents and nursing home patients. In contrast, the United Kingdom adopted a community‐wide sampling approach, sequencing a high percentage of cases across diverse demographics to provide a broader epidemiological picture [37]. The United States also targeted the wider community but faced challenges in data coordination across its more decentralised public health system [38]. Singapore's centrally coordinated and focused sampling strategy enabled efficient allocation of resources, such as its prioritisation of variant detection from imported cases, allowing for rapid adaptation of policies, as necessary, to prevent variant introduction and spread.

Finally, Singapore's integration of genomic data with other surveillance indicators, such as wastewater testing and syndromic surveillance, also sets it apart. While the United States and United Kingdom also developed strong wastewater monitoring systems [39, 40], Singapore used the data source to complement human surveillance data in high‐density areas, such as dormitories and residential zones, provided an additional early indicator and better sense of ongoing community transmission. This complementary approach helped to overcome challenges posed by reduced testing and participation in surveillance programmes as the pandemic progressed.

Overall, Singapore's strategy provides insights that may be useful for other countries when faced with similar challenges. By integrating genomic surveillance into existing respiratory infection monitoring frameworks, Singapore was able to maximise resources and avoid duplicative efforts. Additionally, combining rapid testing methods, such as SGTF, with selective WGS offers a potential model for optimising variant detection when sequencing resources are limited. The use of wastewater and syndromic surveillance as complementary tools could also support early detection of variants and community transmission trends in a more resource‐efficient manner. Singapore's experience exemplifies the potential value of a coordinated response to genomic data, providing a practical reference for countries looking to strengthen their surveillance systems within resource constraints and to support timely public health actions during pandemics.

### 4.5 Challenges of COVID‐19 Surveillance

In Singapore, the surveillance programme faced several challenges that impacted its effectiveness. These included limited laboratory capacity, significant time constraints and testing fatigue among healthcare providers and patients. Each of these issues posed unique obstacles that hindered the timely and comprehensive analysis of COVID‐19 samples, thereby affecting public health decision‐making.

Globally, bottlenecks in data processing were consistently highlighted as a significant challenge for genomic surveillance programmes [6, 16]. Locally, the high number of COVID‐19 cases within a short timeframe coupled with limited laboratory capacity for WGS meant that only a fraction of swabs done could be sent for WGS. Additionally, there was the time lag involved in acquiring WGS results. The typical turnaround time for sequencing clinical samples is about 2 weeks, due to limited machines and the time required for sample preparation and sequencing. Consequently, public health decisions often relied on data that were about 2 weeks old. Workflows were also disrupted when unexpected issues occur such as machine errors or maintenance requirements.

To reduce this time lag, NPHL had the capacity to rapidly run PCRs in unique situations or an ad‐hoc basis, such as during the appearance of a new potential variant of concern. MOH also used surrogate laboratory markers such as SGTF to calculate preliminary proportions of COVID‐19 variants, which has a shorter turnaround time of 2–7 days as compared to the usual 2 weeks. For example, in May 2022, we observed a significant increase in the proportion of SGTF‐positive samples, which rose from just 3% of all local samples to surpass the proportion of SGTF‐negative samples by late June, reaching above 60%. This reflected the change in the predominance from the SGTF‐negative BA.2 to the SGTF‐positive BA.4 and BA.5. Subsequently, a gradual transition in SGTF prevalence was observed from late August to September, indicating the increasing dominance of the XBB variant.

This provided preliminary information that policy makers could act on. Furthermore, Singapore's government relied on various data types besides genomic data, including total caseloads, hospitalisation rates and severe case reporting, to gain a comprehensive understanding of the epidemiological situation.

As the pandemic persisted in Singapore, a noticeable fatigue among patients and healthcare providers to participate in ARI surveillance became apparent likely because nasopharyngeal swabs were uncomfortable and COVID‐19 was no longer seen as a threat. Since the submission of swab samples was strongly encouraged but still voluntary on the part of the primary care clinics, the number of samples submitted decreased steadily from hundreds of samples to less than 100 as the pandemic wore on. Sample collection numbers from the community varied from 237 to 920 in May 2022. However, by December 2022, we received only 93 COVID‐19 samples from the same pool, further decreasing then stabilising at 50–60 samples by February 2023. The dwindling numbers of samples presented several challenges for the MOH. First, smaller sample sizes led to greater variation in the composition of each variant within the total data pool. Therefore, the data on variant composition became less accurate and more prone to random sampling errors. Ling‐Hu et al. [6] similarly mentioned the issue of inconsistent and problematic data collection affecting overall genomic surveillance, highlighting that this issue is not unique to Singapore. This emphasises the need for increased engagement and streamlining efforts to ensure adequate and sustainable data collection for genomic surveillance programmes.

### 4.6 Limitations of Surveillance Strategy

Singapore's surveillance strategy had several key limitations. The MOH decided to prioritise identification of COVID‐19 variants that were causing more severe disease, deliberately selecting more of those cases for WGS instead of a purely randomised sample selection. Consequently, this could have opened a gap in our understanding of those causing milder cases. This gap was accentuated by the fact that individuals with mild symptoms may not seek medical attention, thus escaping testing altogether. Given the limitations, this strategic decision was justified, since the variants of concern to public health authorities would naturally be those that are causing more severe symptoms and disproportionately causing increased hospitalisation and death. The loss of data for milder circulating variants may have meant a less complete picture however, and milder cases would still drive up healthcare demand and absenteeism in the workforce [41], thereby creating unanticipated strain on the healthcare resources of a country. A potential solution to this limitation would be to increase the overall sample size to still ensure representation of both severe and milder cases.

As previously mentioned, samples for gathering genomic data were getting increasingly scarce as the pandemic progressed. Testing fatigue aside, patients were more likely to refrain from seeking medical attention for COVID‐19 infections as the virus became more endemic [42]. As such, most of the cases being sampled by our clinics were people who had specific reasons to present to a doctor, such as being frailer and having more severe symptoms [43]. This may have further skewed the results of the genomic surveillance programme, as a large proportion of patients with mild or no symptoms would not have been sampled.

Together, the aforementioned limitations may have reduced the comprehensiveness of the variant analysis and skewed the data by underrepresenting milder cases or less severe variants circulating in the community. To mitigate such challenges in future surveillance programmes, strategies could include increasing engagement with the public and healthcare providers to sustain testing participation, or exploring the use of incentives for increasing sample collection. Additionally, applying sampling adjustments or weighted analyses could help account for demographic or case‐severity biases in future analyses, thereby ensuring more accurate representations of variant trends.

We earlier alluded to wastewater surveillance as an alternative strategy to human testing. Many countries adopted this method, allowing authorities to collect crucial COVID‐19 transmission data and genomic surveillance [44]. Similarly, Singapore's National Environmental Agency (NEA) led efforts to conduct wastewater genomic surveillance [29, 45] of COVID‐19 variants shed into the country's sewage system. This allowed for complementary detection of circulating variants in the community that were otherwise not picked up in human surveillance. Though this strategy helped the MOH to gain a better overall picture of the spread of COVID‐19, wastewater genomic surveillance produces only ecological data, which could not be linked to demographics and clinical outcomes.

Finally, the complexity of genomic data increased with the coexistence of multiple variants in the community. Earlier on in the pandemic, waves were often caused by a single variant becoming more dominant over the rest. Its rapid spread across the population then caused the respective waves. However, as COVID‐19 became endemic across the world (end 2022 onwards), there were often multiple variants circulating in the community at any one time. This evolution from a single dominant variant picture to a multi‐variant picture made it significantly harder to predict disease trends or anticipate COVID‐19 waves compared to previous times.

Despite these limitations, the effectiveness of global COVID‐19 genomic surveillance is apparent when examining its tangible impacts on policy. Genomic surveillance allows public health officials to track pathogen mutations and new variants, informing them about the infectiousness and efficacy of existing treatments or vaccines. It guides resource allocation by identifying variant concentrations, enabling targeted testing, isolation, and healthcare staffing. This surveillance monitors disease spread and intervention effectiveness, helping to assess and adjust public health strategies. Additionally, it aids in vaccine development by highlighting mutations that may evade current vaccines, facilitating the creation of updated vaccines or boosters.

## 5 Conclusion

The widespread implementation of genomic surveillance during the COVID‐19 pandemic has proven instrumental in tracking the emergence and spread of variants globally. This capability enabled timely responses and informed public health decisions, contributing significantly to the understanding of epidemiological patterns. Singapore's use of this technology exemplifies its effectiveness in a densely populated, high‐traffic environment, allowing for early detection and proactive measures. The relative success of genomic surveillance in managing COVID‐19 highlights its potential for other diseases, emphasising the need for its continued development and deployment to safeguard national, regional and global health security.

## Author Contributions

**Hao Yi Tan:** conceptualization, methodology, writing – original draft, writing – review and editing, project administration, formal analysis, visualization, data curation. **Nur Huda Khamis:** conceptualization, writing – original draft, writing – review and editing, visualization, methodology, formal analysis, project administration, data curation. **Alvin Goh:** writing – original draft, writing – review and editing, visualization, project administration. **Tania K. L. Mah:** writing – original draft, writing – review and editing, visualization, project administration. **Benny Yeo:** methodology, writing – review and editing. **Jie Yin Ngan:** methodology, writing – review and editing. **Yichen Ding:** methodology, writing – review and editing. **Cui Lin:** methodology, writing – review and editing. **Sae‐Rom Chae:** writing – review and editing, supervision. **Phoebe Lee:** writing – review and editing, supervision. **Zheng Jie Marc Ho:** writing – review and editing, writing – original draft, conceptualization, methodology, project administration, supervision.

## Conflicts of Interest

The authors declare no conflicts of interest.

### Peer Review

The peer review history for this article is available at https://www.webofscience.com/api/gateway/wos/peer‐review/10.1111/irv.70060.

## Data Availability

The data that support the findings of this study are not publicly available due to restrictions related to patient privacy and institutional guidelines. Data may be available from the corresponding author upon reasonable request and with permission from the relevant institutional review board.
